# Neurophysiological and clinical outcome measures of the impact of electrical stimulation on spasticity in spinal cord injury: Systematic review and meta-analysis

**DOI:** 10.3389/fresc.2022.1058663

**Published:** 2022-12-16

**Authors:** Sarah Massey, Anne Vanhoestenberghe, Lynsey Duffell

**Affiliations:** ^1^ Aspire Centre for Rehabilitation Engineering and Assistive Technologies, Division of Surgery and Interventional Sciences, University College London, London, United Kingdom; ^2^ Department of Medical Physics & Biomedical Engineering, University College London, London, United Kingdom; ^3^ School of Biomedical Engineering & Imaging Sciences, King's College London, London, United Kingdom

**Keywords:** spinal cord injury, spasticity, transcutaneous spinal cord stimulation, transcutaneous electrical nerve stimulation, functional electrical stimulation

## Abstract

This systematic review and meta-analysis aims to determine whether non-invasive electrical stimulation (ES) is effective at reducing spasticity in people living with spinal cord injury (SCI). PubMed, Web of Science, Scopus and Cochrane Central Register of Controlled Trials databases were searched in April 2022. Primary outcome measures were the Ashworth scale (AS), Modified Ashworth scale (MAS), Pendulum test and the Penn spasm frequency scale (PSFS). Secondary outcomes were the Hoffman (H)- reflex, motor-evoked potentials (MEPs) and posterior-root reflexes (PRRs). A random-effects model, using two correlation coefficients, (Corr=0.1, Corr=0.2) determined the difference between baseline and post-intervention measures for RCTs. A quantitative synthesis amalgamated data from studies with no control group (non-RCTs). Twenty-nine studies were included: five in the meta-analysis and 17 in the amalgamation of non-RCT studies. Twenty studies measured MAS or AS scores, 14 used the Pendulum test and one used the PSFS. Four measured the H-reflex and no studies used MEPs or PRRs. Types of ES used were: transcutaneous electrical nerve stimulation (TENS), transcutaneous spinal cord stimulation (TSCS), functional electrical stimulation (FES) cycling and FES gait. Meta-analyses of 3 studies using the MAS and 2 using the Pendulum test were carried out. For MAS scores, non-invasive ES was effective at reducing spasticity compared to a control group (*p = 0.01*, Corr=0.1; *p = 0.002*, Corr=0.2). For Pendulum test outcomes, there was no statistically significant difference between intervention and control groups. Quantitative synthesis of non-RCT studies revealed that 22 of the 29 studies reported improvement in at least one measure of spasticity following non-invasive ES, 13 of which were statistically significant (*p < 0.05*). Activation of the muscle was not necessary to reduce spasticity. Non-invasive ES can reduce spasticity in people with SCI, according to MAS scores, for both RCT and non-RCT studies, and Pendulum test values in non-RCT studies. This review could not correlate between clinical and neurophysiological outcomes; we recommend the additional use of neurophysiological outcomes for future studies. The use of TSCS and TENS, which did not induce a muscle contraction, indicate that activation of afferent fibres is at least required for non-invasive ES to reduce spasticity.

## Introduction

Spinal cord injury (SCI) is the damage of the spinal cord from trauma or disease (1). This damage can lead to neurological disorders such as spasticity. As well as people with SCI, spasticity also affects those with multiple sclerosis, stroke and traumatic brain injury. Globally, spasticity affects around 12 million people (2). At discharge, there is a prevalence of around 65%, following traumatic SCI (3); for chronic injuries, spasticity affects 61% of people with a motor-complete injury and 79% of people with a motor-incomplete injury (4).

Pharmaceutical intervention, such as the use of botulinum toxin, is a common method of spasticity management (5), however it may cause adverse side-effects such as weakness and drowsiness. Evidence suggests that it may not improve active function, or activities of daily living, particularly for those with upper limb spasticity (6–8).

There is limited recommendation for the use of electrical stimulation (ES) in the clinical management of spasticity, given by the National Institute for Health and Care Excellence (NICE) guidelines in the United Kingdom. Published reports recommending the use of transcutaneous electrical nerve stimulation (TENS) and functional electrical stimulation (FES) for the rehabilitation of people with SCI focus on its use for increasing strength and function, not for spasticity management (9). Spinal cord stimulation (SCS), whether delivered invasively or transcutaneously (TSCS), is not included in the NICE guidelines as a method of spasticity management. Its primary recommendation of use is for pain management (10–12).

With increased spinal excitability being a contributing factor to spasticity, measures of Hoffman (H)-reflex and posterior-root reflexes (PRRs) are ideal for probing changes following intervention (13–15). Alterations in the corticospinal pathway and descending inhibition can be investigated by measuring motor-evoked potential (MEP) amplitude, shown to correlate with severity of spasticity (16, 17). Although it is not commonplace, including these neurophysiological measures to research studies, as well as clinical outcomes, can deepen our understanding of the mechanisms behind clinically important improvements in spasticity, following intervention.This systematic review investigates the effects of non-invasive ES delivered to the peripheral nervous system (including TSCS) on limb spasticity in people with SCI. Meta-analyses aimed to investigate the relationship between clinical and neurophysiological outcomes, following non-invasive ES. Specifically, the primary outcome measures assessed are Modified Ashworth scale (MAS), Pendulum test and Penn spasm frequency scale (PSFS) scores. Secondary outcomes include the H-reflex, PRR and MEP amplitudes.

There are limited randomised-control trials (RCTs) exploring the benefits of non-invasive ES for spasticity in people with SCI. The low number of participants within this cohort does not lend itself to the large-scale studies seen in other research fields.

Existing reviews which analyse similar data include: systematic reviews on the effects of TENS on limb spasticity arising from various prognoses (18); the treatment of functional electrical stimulation (FES) for people with SCI (19); and the effects of ES parameters on lower limb spasticity in people with SCI (20). These provide some evidence into the success in decreasing spasticity in populations with SCI of neuromodulation, such as SCS and TENS without exercise (18, 20–23), as well as with exercise, such as FES cycling and gait (19, 20, 24). However, these reviews do not consider the neurophysiological changes when non-invasive electrical stimulation is used for spasticity management. This present systematic review considered the effects of all non-invasive forms of ES on clinical outcomes of limb spasticity, in people with SCI. It also assessed possible correlations between clinical changes in spasticity and neurophysiological outcome measures.

The aims of this systematic review and meta-analysis are to determine whether: (i) non-invasive ES delivered to the peripheral nervous system can improve MAS, Pendulum test and PSFS scores in people with SCI (ii) there is a specific non-invasive ES protocol which is more effective at reducing spasticity (according to these outcome measures) in people with SCI (iii) there are any correlations between clinical outcome measures of spasticity, such as the MAS, Pendulum test and PSFS score, and neurophysiological measures, such as H-reflex, PRR and MEP amplitude (iv) the current literature allows us to draw clinically meaningful conclusions.

## Methods

This systematic review and meta-analysis has been carried out in accordance with the Preferred Reporting Items for Systematic Reviews and Meta-Analyses (PRISMA) and Cochrane guidelines (25). It has been registered with PROSPERO international prospective register of systematic reviews (registration number CRD42020186215), which was amended in April 2022 from a previous publication (26), to form this systematic review.

### Search criteria

In April 2022, S. Massey performed searches of PubMed, Web of Science, Scopus and Cochrane Central Register of Controlled Trials (Cochrane CENTRAL) databases, using the following search strategy: electric* AND stimulation AND spastic* AND spinal AND (injury OR lesion) AND (Ashworth scale) OR (pendulum test) OR (Penn spasm frequency scale). No restrictions for publication date were used.

### Eligibility criteria and study selection

Search results from all databases were combined in EndNote ×9 software and duplicates were removed. These were then initially screened by title and abstract using the inclusion criteria: (i) human adults with SCI; (ii) provided an intervention of continuous (i.e., excluding TENS and TSCS interventions that were delivered intermittently), non-invasive ES, delivered to the peripheral nervous system, either alone or in combination with movement therapy; (iii) investigating limb spasticity (i.e., exclude studies investigating bladder spasticity); (iv) use of the MAS, Pendulum test and/or PSFS to assess changes in spasticity due to intervention; (v) in English language.

Only RCTs which used the MAS, Pendulum test or PSFS as outcome measures were included in meta-analyses and statistical analysis. Data from studies that only used an intervention group (i.e., observational or case studies), which used the same outcome measures, were collated and reviewed but not statistically analysed.

It should be noted that neurophysiological outcomes were considered to be secondary outcomes, and so articles were not screened for their inclusion.

### Methodological quality

Methodological quality of RCTs was assessed using the Physiotherapist Evidence Database (PEDro) scale (27). This included assessment of studies which had more than 1 intervention group, where other groups either received an alternative intervention, or were control groups. Each study was given a score of 1 if it was completely clear that they satisfied the criterion; if it was not explicitly stated, they did not receive the point. Where the analysis of an outcome measure in a study was assessed, only outcome measures relevant to this systematic review were assessed.

Heterogeneity of RCTs was measured using the *I*^2^ statistics when using a random-effects model. Due to the low number of RCTs included in this systematic review, heterogeneity of studies was considered for *I*^2^ > 30% and for *p < 0.10* (25). If studies were deemed to be heterogenetic, sources of variation between protocols (e.g., methodological and statistical diversity) were assessed.

### Outcome measures

The primary outcome measures for meta-analyses were the MAS (including the AS), the Pendulum test and the PSFS. The secondary outcome measures are the H-reflex amplitude, H_max_/M_max_ ratio, PRR and MEP amplitude. For meta-analysis, these measures were only considered if the study also includes one of the primary outcome measures, to allow for effective comparison of clinical and neurophysiological measures of spasticity.

### Data extraction and management

Data extracted from included studies were methodological design; number of participants; ASIA Impairment Scale (AIS); time since SCI; ES location; ES parameters (frequency, pulse width and duration of stimulation). Studies which used ES in a single session only were defined as “acute” studies, and those which delivered ES in multiple sessions were defined as “long-term” studies. Depending on the methodological design, studies were categorised as either RCTs (including crossover designs) or non-RCTs (i.e., case studies and observational studies with no control group comparator).

Data of ES parameters, for studies which used a single frequency which was stated, were plotted using SankeyMATIC (28). Frequencies and pulse widths used were split by type of ES delivered for each paper (FES, TENS, TSCS). TENS interventions were interventions where ES was delivered over a muscle or nerve, with no movement involved, and FES interventions were paired with functional movement.

For studies who used compound scoring for the MAS scores (i.e., the MAS score measured for each individual muscle was summed), if the raw data for individual participants were available, this data was divided by the number of muscles measured, from which the mean and SD was calculated.

For studies which presented separate results for left and right limbs, or for several muscles, the mean and SDs of these results were combined within each study, in accordance with the Cochrane guidelines (25).

For analysis of the acute effects of non-invasive ES on the MAS score, the mean change in score was calculated for each study. The SD of this change was then calculated as the propagation of error (Equation 1, where Corr is the correlation coefficient and 1≤Corr≤−1).

For all meta-analyses carried out, the exact value of the correlation coefficient was unknown due to the nature of the data collected. Therefore, analysis was carried out under the assumptions that Corr=0.1 or Corr=0.2. These values were selected since as Corr→1, it can be assumed that the baseline score had an effect on the post-intervention score. We therefore tested for values Corr>0. We did not test for values Corr>0.2 since, for the data presented here, the value within the square root would be <0.


$$SD_{change}=\sqrt{SD_{baseline}^{2}+SD_{\;final}^{2}-(2\times Corr\;\times SD_{\;final}})$$

### Statistical analysis

Meta-analyses of RCTs were carried out using Review Manager 5.4 software. Analyses of the mean differences of continuous outcome measures were carried out using a random-effects model, using 95% confidence intervals. The mean difference was calculated as the change from baseline to the post-intervention value. A minimum of two studies were required to carry out this analysis. Where a single study investigated several non-invasive ES interventions, the same control group was used as the comparator for each intervention. Statistical significance was considered for *p < 0.05*.

For analysis of non-RCTs, studies were grouped depending on their outcome measure and type of trial (single-session (acute) or multi-session over several days (long-term)). Results for individual participants in each study were amalgamated and graphically presented. Data from studies presented as mean ± SD were also included if data for individuals was not available.

Studies were included in one type of analysis only. If a study qualified to be included in the RCT meta-analysis, it was not included in results for non-RCT studies.

## Results

### Study selection

**Figure 1:**
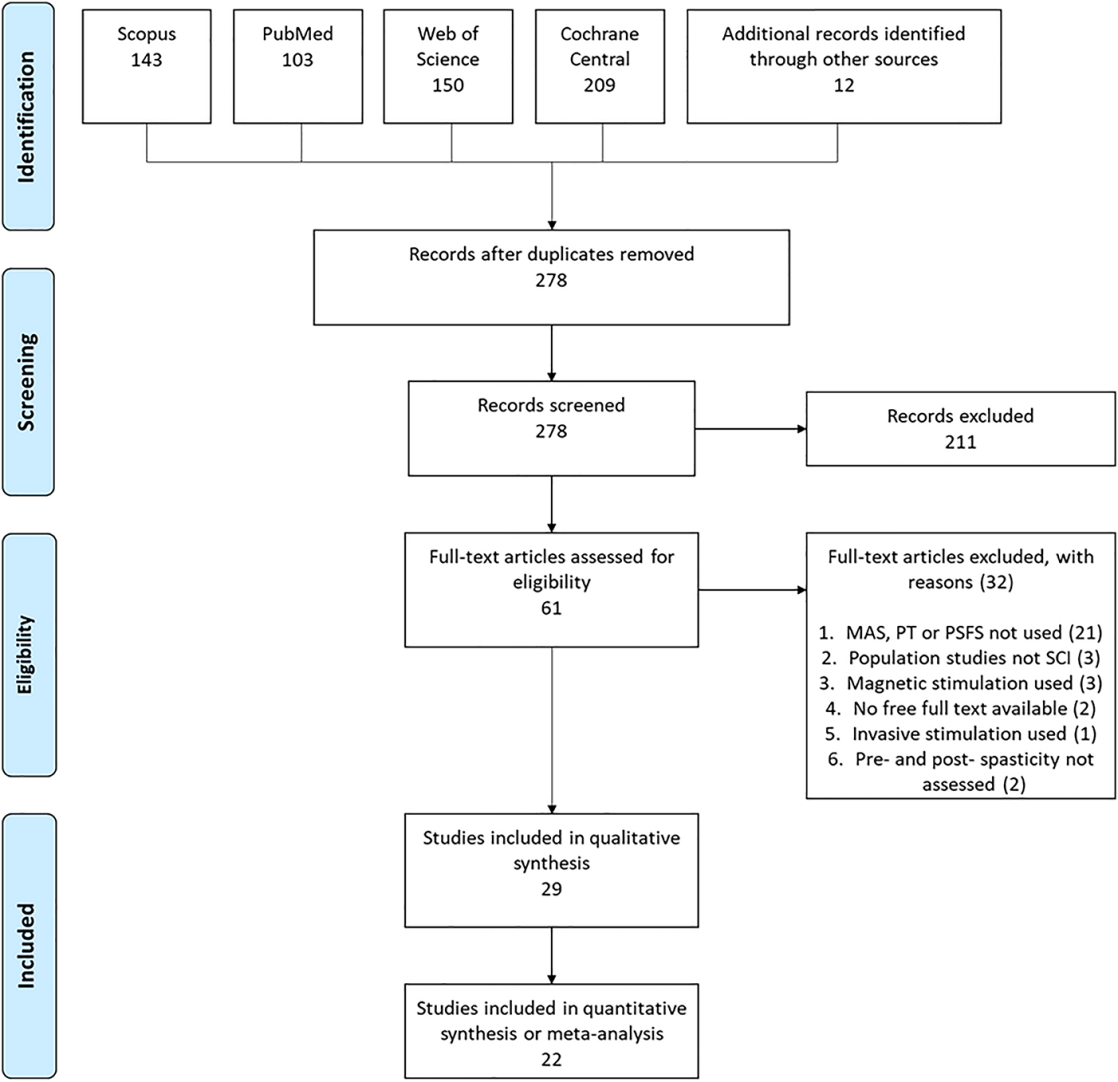
PRISMA flow diagram of included studies.

A total of 605 papers were identified through database searches. A further 12 papers found through personal databases. A PRISMA flow diagram is presented in Figure 1. Once duplicate papers had been removed, 278 papers were screened by their title and abstract and 211 were removed as it was clear that they did not meet the inclusion criteria. Sixty-one full-text articles were reviewed, and 32 were removed (see Figure 1 for the reasons for exclusion). This left 29 studies to be included in the systematic review. Of these, 5 studies were included in the meta-analysis and 17 in the amalgamation and presentation of results from non-RCT studies. The remaining 7 studies were not included in the meta-analysis nor the amalgamation, either because results were not presented or because the outcome measures presented were not consistent with the other included studies.

### Included studies

Of the 29 studies included in this systematic review, the spread of interventions used was as follows: 10 studies assessed the effects of TENS on spasticity (9 for the lower limbs and 1 for the upper limbs) (29–39); 6 used TSCS, all targeting the lower limbs (40–45); 9 used FES cycling (46–53); 3 used FES gait (54–56); and one assessed FES for the lower limbs as well as TENS (already counted) (37).

It should be noted that the study by Mazzoleni et al. (51) studied the effects of 20 sessions of FES cycling followed by 20 sessions of robot-assisted FES gait training. For the purposes of this systematic review and meta-analysis, results were only taken from the FES cycling phase of this trial, since the cycling phase may have influenced results obtained during the subsequent FES gait phase.

Of the 20 studies that used the Ashworth Scale, three used the AS scoring (0, 1, 2, 3, 4) and 17 used the MAS scoring (0, 1, 1+, 2, 3, 4). Of these 17, 4 studies used compound scoring, two studies assigned the 1 + score the value of 1.5 and one study assigned 1 + score with 2, 2 with 3 and so on. Of this 20, 8 were included in the quantitative synthesis; three were RCTs and 13 were non-RCTs. Four studies were excluded from these analyses: three because data were not presented (31, 54, 55), and one because compound scoring was used and averaged across participants (51). In these cases, the author was contacted to request access to their data, however this was not successful for all studies.

Fourteen of the included studies used the Pendulum test to measure changes in spasticity. Of these 14 studies, 8 reported Pendulum test results as the Relaxation Index (R_2n_) [described by Bajd and Vodovnik (57)] (30, 44, 47, 54, 42, 58) [with one reporting results as the change in R_2n_ (59)], 4 as the first swing excursion (40, 41, 45, 52), [defined as “the angle at which the movement of the lower leg reverses from flexion to extension after release of the heel” (40)]. One study only reported the statistical analysis of their Pendulum test results (55), and the remaining study did not report their results as they were statistically non-significant (32).

The PSFS was included in one study as an outcome measure, however it was reported as a single value and it was not clear whether the two components of the PSFS were averaged together (51).

Four studies included the H-reflex as an outcome measure (29, 33, 52, 60). All studies reported their results as H_max_/M_max_. No studies included in the final systematic review included MEPs or PRRs in their outcome measures.

### Methodological quality of RCTs

Assessment of randomised trials (whether the group comparator was a control group or assessing another form of non-invasive ES) is shown in Supplementary Table S1.

It should be considered that, due to the nature of non-invasive ES as an intervention, it was noted by most assessed studies that blinding the participant and the therapist who administered the intervention was not possible. For some studies, one assessor was blinded and others were not (34, 47).

### Participants

A total of 421 participant experiences of people with a SCI were included in this systematic review. Seventeen studies included people with AIS A SCIs and 8 included people who had incomplete injuries. Ten studies included participants that were >1-year post-injury, three studies included participants that were >6-months post-injury, and 9 studies included at least one participant who was <6-months post-injury. A summary of these parameters is given in Supplementary Table S2.

Only 11 studies included information on whether participants were permitted to continue taking anti-spasticity medication (34, 37, 40, 42, 43, 45–47, 60–62). In one of these 11 studies, taking anti-spasticity medication was an exclusion criteria (47), whilst the 10 other studies allowed participants to continue taking their medication.

### Stimulation parameters

**Figure 2:**
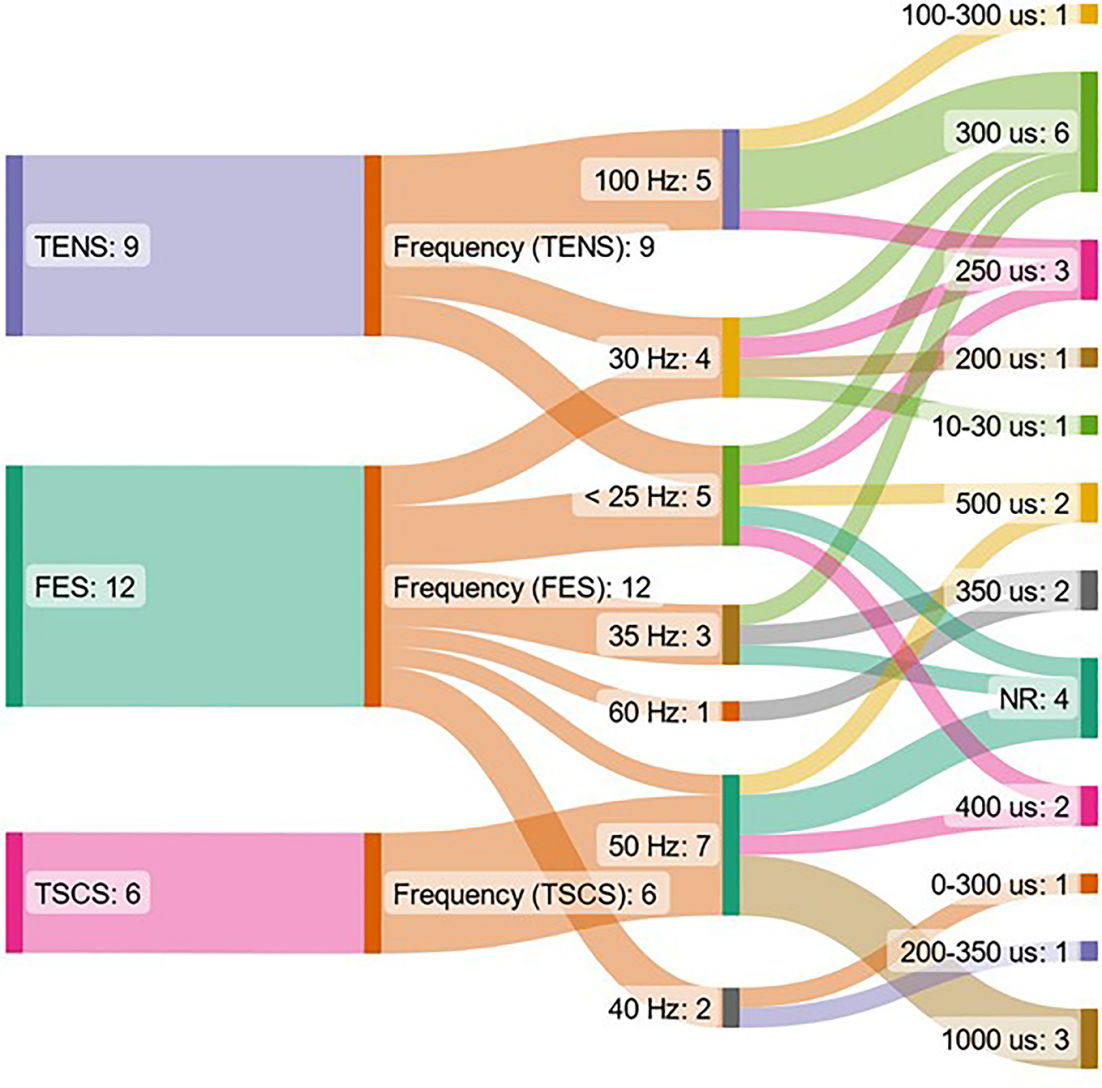
Representation of the form of non-invasive ES, frequency and pulse width used in included studies. The figure shows data from papers which used a single stimulating frequency which was reported only. ES = electrical stimulation, FES = functional electrical stimulation, NR = not reported, TENS = transcutaneous electrical nerve stimulation, TSCS = transcutaneous spinal cord stimulation.

The range of ES frequencies used in studies included in this systematic review was 5–1000 Hz. The range of pulse widths used was 0.01–100 ms. One study investigated the effects of TENS delivered with varying frequency/pulse width (ms) pairs, which were as follows: 100/0.1, 100/0.01, 100/1, 1000/0.1, 1000/0.01, 10/0.1, 10/0.01, 10/1 (59). The distribution of frequencies and pulse widths used in studies which used a single frequency value, which was stated, is shown in Figure 2. This shows that there is more variation in pulse widths used between studies compared to frequency, and that the pulse width used does not necessarily correspond to a particular frequency used. There was a larger variation of frequencies used for FES interventions compared to TENS and TSCS. All TSCS interventions used a 50 Hz frequency.

The range of ES amplitude reported was 8–160 mA (discounting studies which reported ES amplitude as “up to”, and Krause et al. (47), as their reported stimulation intensities included their passive cycling arm (0–99 mA)). Reported methods of determining ES amplitude included: below motor threshold (MT); producing paraesthesia (40–42, 45); highest tolerated intensity (53); 2× sensory threshold in healthy, able-bodied participants (31, 33); sub-PRR threshold (43); at MT, or below when muscle spasticity occurred (34); 0.9× lowest MT in all stimulated muscles (44); 3× MT (37, 60); and 0.8× MT (60). Where studies did not report a method for setting the stimulation amplitude for each participant, they stated the value of stimulation amplitude. Supplementary Table S2 shows a summary of all stimulation parameters of included studies.

### Summary of results from included studies

Overall, 22 of the 29 studies included in this systematic review reported an improvement in at least one measure of spasticity following non-invasive ES, 13 of which were statistically significant changes (*p < 0.05*), either from their baseline measure, or when compared to a control group (see a summary of all results in Supplementary). Twelve studies found no change in at least one included outcome measure and two studies reported a possible, non-statistically significant, worsening of spasticity (52, 62).

### Meta-analysis of RCTs

This systematic review includes results from three RCTs which used the MAS as an acute measure of spasticity (47, 49, 60). All studies used a crossover design. The study by Van der Salm et al. (60) compared three forms of TENS, delivered to tibialis anterior, triceps surae, and the S1 dermatome (delivered over the side of the foot), to a control intervention. The study by Krause and Straube (47) compared the effects of FES cycling with passive cycling and Ralston et al. (49) compared FES cycling to no FES cycling.

**Figure 3:**
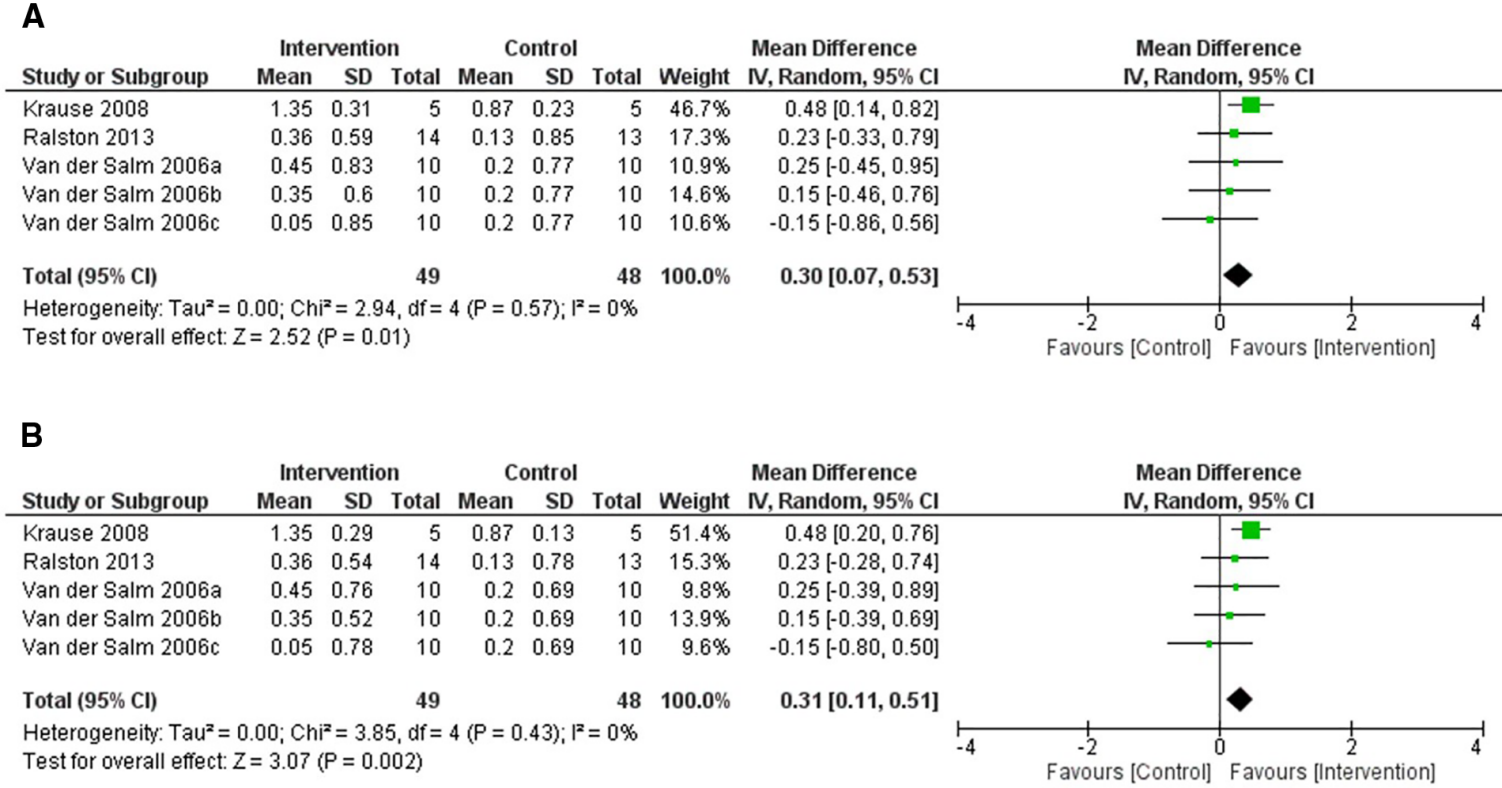
Forest plot using a random effects model of RCT studies using the MAS to test for the acute effects of non-invasive electrical stimulation. 2006a-c represent the 3 interventions that were tested in the single study performed by Van der Salm et al. (2006): TENS delivered to the agonist, antagonist and the S1 dermatome respectively. The black diamond represents the average effect of non-invasive electrical stimulation. (**A**) shows results for Corr = 0.1 and (**B**) for Corr = 0.2. CI = confidence interval, MAS = modified Ashworth scale, RCT = randomised-control trial, TENS = transcutaneous electrical nerve stimulation.

Figure 3 shows that the results from the included studies favour the intervention for both Corr=0.1 (*p = 0.01*) and Corr=0.2 (*p = 0.002*). In both cases, FES cycling delivered by Krause and Straube (47) showed the largest change in MAS score (*N* = 5), which was the most heavily weighted result in this random-effects model. Excluding S1 dermatome stimulation (60), mean differences in MAS scores for all other forms of non-invasive ES showed an improvement.

There were two RCTs which included the Pendulum test in its outcome measures (40, 41). One trial used a cross-over design (40), which compared the effects of TSCS to sham stimulation. The other was a RCT, which compared TSCS to sham stimulation in a separate control group (41).

**Figure 4:**
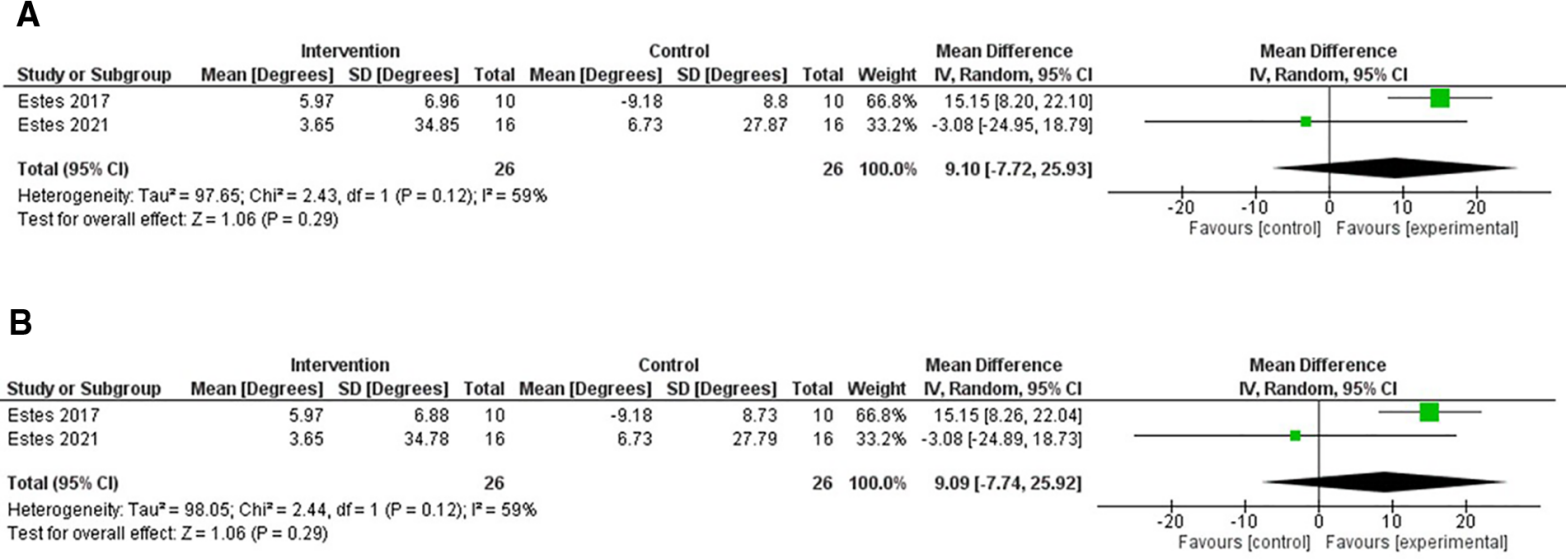
Forest plot using a random effects model of RCT studies using the FSE. The black diamond represents the average effect of non-invasive electrical stimulation. (**A**) shows results for Corr = 0.1 and (**B**) for Corr = 0.2. CI = confidence interval, FSE = first swing excursion, RCT = randomised-control trial.

Figure 4 shows forest plots for the effects of TSCS vs. a sham-control for these RCTs. On average, the two studies are in disagreement with one another, with the 2017 Estes et al. study (40) showing an overall improvement in spasticity, whereas the 2021 Estes et al*.* study (41) demonstrated varied results, which overall favoured the control group.

### Acute changes in MAS score in non-RCT studies

**Figure 5:**
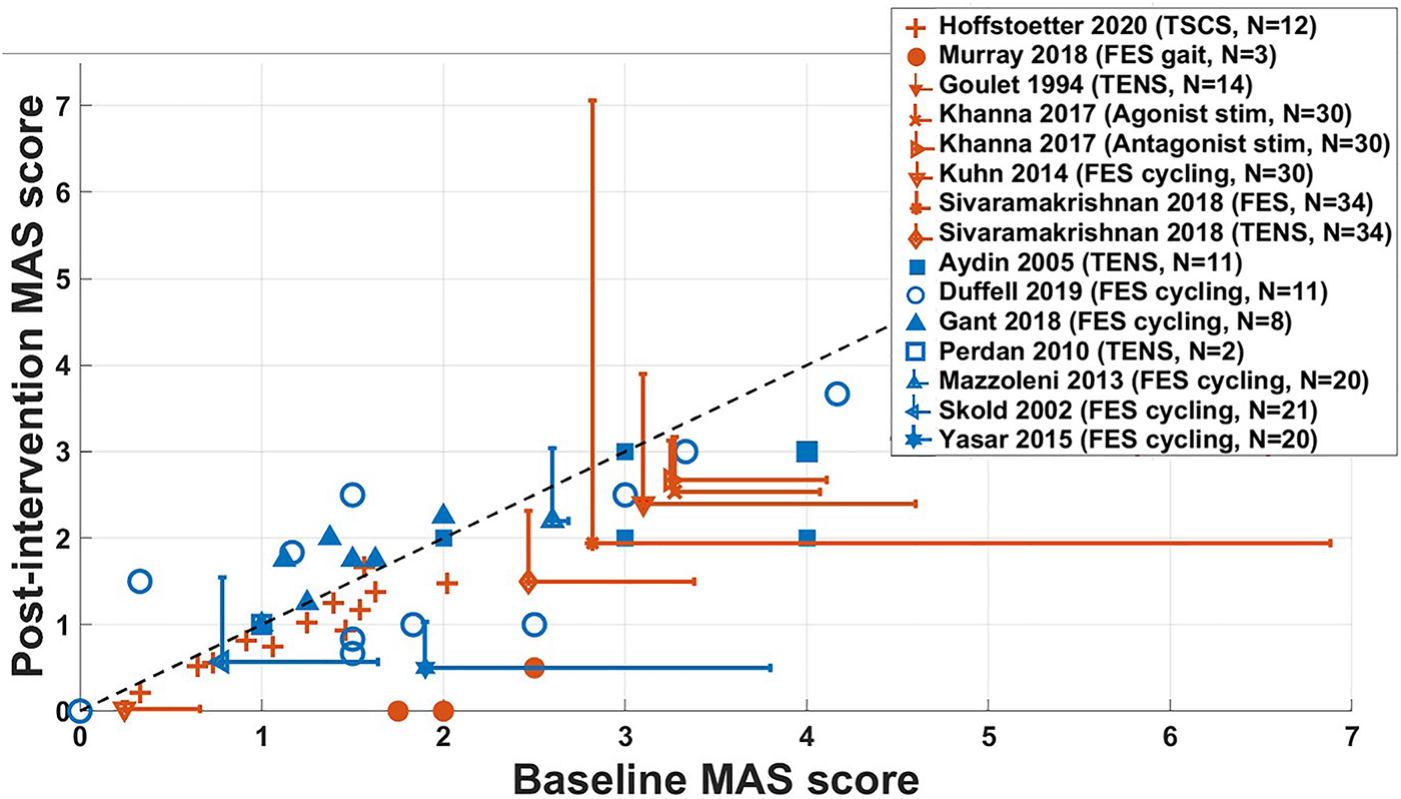
Changes in the MAS score of acute (orange, *N* = 112) and long-term (blue, *N* = 93) non-RCT studies. Datapoints with error bars represent studies where data has been presented as mean ± SD and those without show results for individual participants. The dashed line indicates no change in MAS score following intervention. Results for Sköld et al. (2002) represent the combination of averaged MAS scores in knee flexors and extensors, in left and right legs, which were not measured in every participant, giving *N* = 21. For Yaşar et al. (2015), averaged MAS scores were measured in knee flexors and extensors for all participants, giving *N* = 20. Results for Sivaramakrishnan et al. (2018), spasticity was measured in the hip adductors in 19 legs across 10 participants, in 11 legs for the knee extensors and 4 legs for plantar flexors, giving *N* = 34. FES = functional electrical stimulation, TENS = transcutaneous electrical nerve stimulation, TSCS = transcutaneous spinal cord stimulation.

Baseline and post-intervention changes from both acute (orange) and long-term (blue) non-RCT studies using the MAS score are shown in Figure 5; data from studies for individual participants (29, 35, 43, 53, 56) and averaged results (33, 34, 48, 50, 37) are shown. Datapoints below the dashed line show results for which the MAS score was reduced following intervention. For long-term studies, only baseline measures and results measured after the last intervention session are presented.

This figure shows that the included acute studies were, on average, more effective at reducing spasticity immediately following the intervention, compared to long-term studies. Studies by Khanna and Kaur (34) and for the TENS intervention by Sivaramakrishnan et al. (37), showed a decrease in spasticity in all participants. Averaged results in studies by Goulet et al. (33), and for the FES intervention used by Sivaramakrishnan et al. (37) show large standard deviations, indicating a large variance across results for individual participants, where some may not have benefitted from the intervention. For participants who took part in the study by Gant et al. (52), MAS scores of all participants were either increased or unchanged following 19 weeks of FES cycling.

### Acute changes in the R_2n_ in non-RCT studies

**Figure 6:**
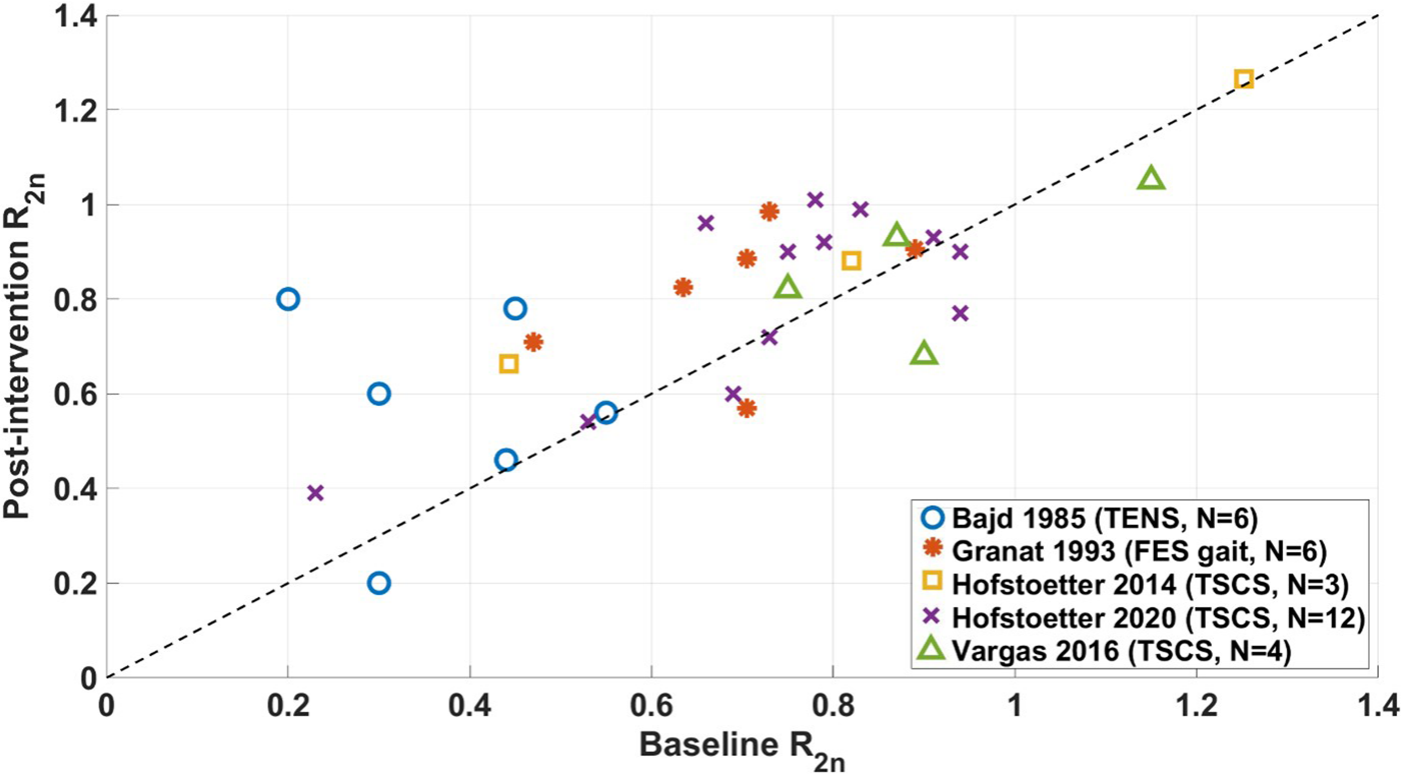
Changes in the R2n of non-RCT studies. Datapoints along the dashed line signify no change in R2n following intervention. FES = functional electrical stimulation, TENS = transcutaneous electrical nerve stimulation, TSCS = transcutaneous spinal cord stimulation.

R_2n_ data from non-RCT studies is shown in Figure 6. All datapoints are from studies for which Pendulum test results for individual participants were available and reported as R_2n_ (30, 44, 54, 42, 58). Points above the dashed line show where R_2n_ measures have increased, indicating a decrease in spasticity.

For the majority of studies, the intervention decreased the level of spasticity measured by R_2n_ (30, 54, 42, 58). Results reported by Vargas Luna et al. (44) showed that 1 out of 4 of their participants had worsened spasticity, 2 had improved spasticity, and the authors determined that one participant did not have spasticity at baseline, since their value of R_2n_∼1.

The most marked changes were in Bajd et al. (30). In their study, participants had a higher level of spasticity at baseline compared to other studies, which may have contributed to the larger reported change following intervention. One quarter of the participants in the study by Hofstoetter et al. (58) had improved spasticity following TSCS, while 4 had unchanged or worsened spasticity.

### Changes in the H_max_/M_max_ ratio in non-RCT studies

**Figure 7:**
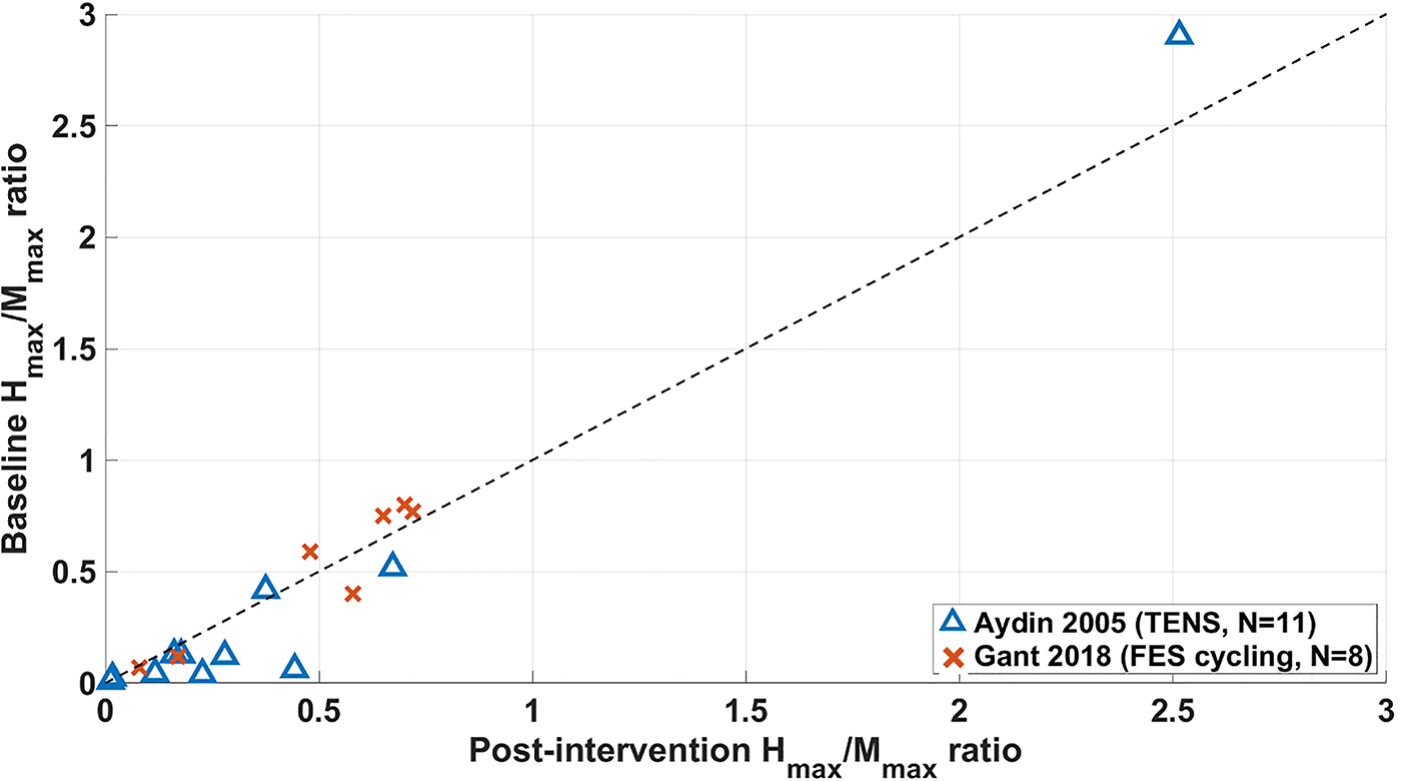
Changes from baseline to end-of-intervention in Hmax/Mmax ratio of long-term non-RCT studies. FES = functional electrical stimulation, TENS = transcutaneous electrical nerve stimulation.

Results from non-RCT studies which used the H_max_/M_max_ ratio as an outcome measure are shown in Figure 7 for studies where results for individual participants were available.

Seven participants in the study by Aydin et al. (29) and two in Gant et al. (52) showed a reduction in H_max_/M_max_ ratio from their baseline measure to the final measure (the follow-up measure for Aydin et al.), which evidence suggests is characteristic of a reduction in spasticity (13–15, 63). However, remaining participants showed either no change or an increase from their baseline measure, indicating that these interventions had either no effect or a negative impact on the majority of participants presented in Figure 7.

Average changes of H_max_/M_max_ ratio of −0.06 (29) and 0.23 (52) from baseline to the end-of-intervention stage were calculated. When comparing this data with that shown in Figure 5, MAS scores in the Aydin study showed that 9 of 11 participants had an improved MAS score and 2 unchanged, with an average change of 0.64. In the study by Gant et al. (52), 5 of 8 participants had worsened MAS scores and 3 unchanged, with an average change of 0.23.

### Other reported outcome measures

Most studies included in this systematic review also reported benefits to their participants in measures other than those required for eligibility in this review. Aydin et al. (29) and Yaşar et al. (64) found statistically significant reductions in the Functional Independence Measure following TENS and FES cycling respectively. Clonus scores were reduced in Goulet et al. (33) and Oo et al. (31) following TENS, and in Hofstoetter et al. (43) following TSCS. A summary of changes in all reported outcome measures is shown in Supplementary Table S2.

## Discussion

### Summary of findings

Results from this systematic review showed that changes in spasticity using MAS scores demonstrate an overall effectiveness of non-invasive ES compared to a control group. However this is not the case with the Pendulum test. Results from non-RCTs overall showed a reduction in spasticity immediately following non-invasive ES. An overall reduction in spasticity measured by MAS scores was also seen in long-term trials. However, there was one study in these results where a 19-week programme of FES cycling caused either an increase or no change in MAS scores (52), as well as 4 cases where spasticity remained unchanged at the end of the study, compared to baseline measures (46, 48, 49, 62). When measured by the first swing excursion, there was no clear improvement in spasticity following TSCS, with a decrease in spasticity in one study (40), but not the other (41).

In non-RCT studies, most participants benefitted from non-invasive ES where spasticity was measured by the Pendulum test. Only Gant et al. (52), reported no change in the Pendulum test following intervention (FES cycling), the same study also reported no changes or a worsening of MAS scores.

Only 4 studies in this systematic review included the H-reflex as a neurophysiological outcome measure (29, 33, 52, 60). Of these, only one reported a reduction in the H_max_/M_max_ ratio from baseline (29), the remaining 3 reported no change, or varied results between participants.

The following sections discuss: variations in stimulation protocols, the impact of the variation in reported outcome measures, any correlation found between clinical and neurophysiological outcomes, the limitations in this systematic review and meta-analysis, and recommendations to future studies investigating non-invasive ES on spasticity in people with SCI.

### Variation between intervention protocols

Although this systematic review covered only 4 methods of non-invasive ES (TENS, TSCS, FES cycling and FES gait), there exist many differences between protocols. Across all interventions, these differences were the frequency, pulse width, duration of intervention, stimulation intensity and the method of determining stimulation intensity.

With the exception of two studies (52, 62), overall, FES interventions tended to reduce spasticity. Similarly, with TENS and TSCS interventions, spasticity was generally reduced across the included studies. This shows that the efficacy of non-invasive ES in reducing spasticity may not be dependent upon its pairing with movement, the frequency, pulse width, or intensity of stimulation.

#### A huge variation in frequency and pulse width parameters is seen across the included studies

For studies included in this systematic review, those which used an FES intervention used a median frequency of 34 Hz and stimulation intensity of 130 mA, whereas TENS and TSCS forms of intervention used a median frequency of 50 Hz and median stimulation intensity of 50 mA; all forms of intervention used a median pulse width of 0.3 ms (see Table 1). Here, FES was given with lower frequencies and higher stimulation intensities compared to TENS and TSCS. These parameters may have been selected so that, at a minimum, an isometric muscle contraction was achieved, and at a maximum, muscle fatigue is minimised. For TENS and TSCS, there is a much larger range of frequencies used, however generally, most studies used frequencies >30 Hz (see Supplementary Table S2 and Figure 2).

**Table 1:** Median (range) of frequencies, pulse widths and stimulation intensities used by all studies which use FES and TENS or TSCS. Where studies report a range of frequencies or pulse widths being used, the average has been taken. Where studies reported a range of stimulation intensities, the maximum intensity was used to account for interventions which included sham stimulation or passive movement (i.e. used 0 mA). In the case of stimulation intensity, only 16 studies reported a quantitative value (see Supplementary Table S2). These values exclude those studied by Vodovnik et al. (1987) due to the large variation in frequencies and pulse widths investigated.

	FES interventions	TENS/TSCS
Frequency (Hz)	34 (20–60)	50 (5–100)
Pulse width (ms)	0.3 (0.05–0.5)	0.3 (0.3–1)
Stimulation intensity (mA)	130 (75–140)	50 (15–160)

FES = functional electrical stimulation, TENS = transcutaneous electrical nerve stimulation, TSCS = transcutaneous spinal cord stimulation.

Research carried out by Vodovnik, Stefanovska and Bajd (59), compared varying combinations of frequencies and pulse widths, at similar stimulation intensities (up to 30 mA) during TENS (10–1000 Hz; 0.01–1 ms—see Supplementary Table S2). This showed that spasticity assessed by the R_2n_ was reduced in 6 out of 7 participants for all variants of combinations, with 100 Hz having the largest effect. However, this was the only study included in this systematic review which directly compared several frequencies and pulse widths for the same form of non-invasive ES and no statistical analysis directly compared the efficacy of different frequencies at reducing spasticity.

From this systematic review, 5 of 6 studies using TENS, which obtained statistically significant improvements in spasticity, used 99 or 100 Hz (29–31, 33, 37), and the other used 30 Hz (34). Of the remaining 4 studies which used TENS [excluding Vodovnik*.* Stefanovska and Bajd (59)], all frequencies used were ≤50 Hz; these studies reported reductions in spasticity that did not reach statistical significance and, in one case, spasticity was worsened (62).

Other review papers share varying opinions on the topic; with some suggesting that frequencies of ∼100 Hz are more effective at reducing spasticity in other aetiologies (21, 22) and others suggesting that TENS delivered at 4 Hz is superior to TENS at 25 Hz in spasticity arising from varying aetiologies (18). In an educational review, it has been suggested that SCS and TSCS at kHz frequencies may be the future of spasticity management (23). Clearly not enough is yet understood to determine which stimulation parameters may be optimal for spasticity management in those with SCI. However, from this systematic review, results suggest that 100 Hz TENS may be superior to frequencies ≤50 Hz for spasticity in SCI.

#### Reduction in spasticity seen across many different ES protocols may not be dependent on stimulation intensity

For FES, participants must sustain a useful muscle contraction. TENS or TSCS is expected to at least activate afferent fibres, with some studies eliciting muscle contractions during intervention and others not. Some studies used a fixed current amplitude across all participants and did not make it clear whether this amplitude caused muscle contraction or not (29, 33, 62). Results presented in Figures 3–7 do not reveal distinct differences between FES, TENS and TSCS protocols.

It is not clear whether the use of sub- or supra- MT stimulation is superior in the reduction of spasticity from the results presented in this meta-analysis. Spasticity may reduce following ES because of complex neural interactions, perhaps involing presynaptic inhibition, recurrent inhibition and/or reciprocal inhibition, all of which rely on the activation of afferent fibres (65). Stimulation of the afferent fibres using sub-MT TENS has been shown to increase feedback to the motor pathways, causing a decrease in excitability of the reflex system (66). However, when ES is delivered supra-MT, antidromic firing, due to the stimulation of efferents, may block afferent feedback (65, 67), meaning that the neural interactions may be even more complex. It is also possible that reduced spasticity following supra-MT stimulation or FES may also occur due to acute fatigue. Results from this systematic review show that both sub- and supra-MT ES reduced spasticity. This suggests that the activation of efferent fibres using ES may not be necessary for reducing spasticity in people with SCI.

Krause, Szecsi and Straube (68) found a statistically significant reduction in spasticity following FES cycling compared to passive cycling alone in five AIS A participants. Similar results were also reported in seven AIS A SCI participants in a non-RCT study by Mazzoleni et al. (51). There were no neurophysiological outcome measures in either study and both measured pre-post changes (in a single-session study (68) and multi-session study (51)). It cannot, therefore, be determined whether spasticity reduced due to plastic changes, or due to fatigue.

Improvements in function due to lower limb FES training following SCI may occur due to the stimulation of central pattern generators (CPGs) (19). Stimulating CPGs may promote increased control of spinal reflexes and therefore increased voluntary control (69). This may also result in a reduction in involuntary muscle spasms. The stimulation of CPGs has not yet been shown to improve spasticity, however, patterned ES delivered to the common peroneal nerve at 100 Hz (selected to reflect firing frequencies during walking) was shown to increase reciprocal inhibition in healthy, able-bodied participants (70). This theory may be a factor influencing the reduction in spasticity, as the stimulation of afferents targets these CPGs.

### Variation in reporting of outcome measures

Studies were included in this systematic review if they used the MAS, Pendulum test or PSFS. However, these measures were not reported in the same manner across studies. For the MAS, studies reported results using compound scoring (the sum of MAS scores across muscles), the average score across several muscles, or for individual muscles or participants. For the Pendulum test, studies reported results as the first swing excursion, or as the R_2n_. In some cases, studies did not report the values of these outcomes if their results were non-significant.

This variation in the reporting of outcome measures in research studies reduces the accuracy and precision of meta-analyses. Seven studies which qualified for this systematic review were not included in the meta-analysis because their results were reported in this way, and they would not share their original data. Future studies should report their outcome measures for individual participants, or ensure that this data is available for authors of future systematic reviews.

### Correlation between clinical and neurophysiological outcome measures

The H-reflex, and therefore spinal excitability, should correlate with spasticity; however much of this evidence exists for people with spasticity arising from a stroke (13–15). One acute study in this systematic review did not find any correlation between H_max_/M_max_ ratio and MAS scores and were only able to elicit H-reflexes in five of 14 participants (33). Another (29) found statistically significant decreases in both AS scores and the H_max_/M_max_ ratio. However, this dual reduction was not seen in the study by Gant et al. (52), following FES cycling, with results varying between participants and time points.

Gant et al. (52) reported an increase in lower limb muscle strength as well as an increase in M_max_ across most participants. If M_max_ were to increase with strength, this may have led to the unclear change in the H_max_/M_max_ ratio and the overall increase in MAS scores. These changes to the muscles, occurring across a long-term training protocol (19 weeks), may influence the variation in spasticity outcome measures.

Investigating both clinical and neurophysiological measures in people with SCI may provide an insight into the mechanisms occurring when a clinically meaningful reduction in spasticity is obtained. This would allow for more studies in people with an intact CNS to draw more meaningful conclusions. Developing these protocols in studies with healthy, able-bodied participants allows for refinement of neuromodulation protocols, including effective frequencies, pulse widths and stimulation intensities. The large variation in the stimulation parameters in this systematic review highlights the need for such studies.

### Limitations

The most important limitation of this systematic review and meta-analyses is the reliance upon data from non-RCT studies to come to the conclusions drawn here. Meta-analyses should be performed on RCT studies since these are least likely to be biased (25). Although the evidence suggests that non-invasive ES is effective at reducing spasticity measured by MAS and Pendulum test scores, 28 of the 29 included studies were targeting lower limb spasticity, with only one paper reporting on two case studies using whole-hand TENS (35). This is partially due to the inclusion of the Pendulum test as one of the primary outcome measures in this systematic review. However, the MAS is commonplace for measuring upper limb spasticity as well as lower limb. There is a clear lack of studies investigating the effects of non-invasive ES in the upper limb, compared to the lower limb. Although some mechanisms may be similar for reduction of spasticity in both upper and lower limbs, conclusions from this systematic review draw on evidence from studies investigating lower limb spasticity.

Data presented in the meta-analysis of RCTs, which used the MAS score as an outcome measure, counted the study by Van der Salm et al. (60) three times. This is not recommended by the Cochrane resources (25), but it was necessary to assess each intervention used in the study separately. An alternative method would have been to combine the effects of the three forms of TENS used. Since the aim of this meta-analysis was to compare the efficacy of various non-invasive ES protocols, this was not appropriate.

There are fewer RCTs on ES for spasticity arising from SCI compared to stroke. In a systematic review by Mahmood et al*.* (22), seven RCTs were included in meta-analysis and they were able to draw conclusions on: appropriate duration of intervention, frequency of intervention and electrode placement. This range of evidence does not exist in RCTs for the SCI population, where studies do not report their outcome measures consistently, making it difficult to draw similar conclusions.

### Recommendations for future studies

This systematic review and meta-analysis have shown that the disparity between research protocols and reporting practices leads to a lack of specificity when amalgamating the data available in this field. Future research protocols should adhere to a standard, using common clinical outcome measures such as the MAS and R_2n_ values scores, making this data readily available as Supplementary Material when it is inappropriate to publish all data.

Future studies should also consider the addition of neurophysiological outcome measures. This component of spasticity measurement may allow for a deeper understanding of the location and neural pathways involved in spasticity modulation following non-invasive ES. By understanding which pathways may contribute to a reduction in spasticity, our understanding of the effect of varying the stimulation intensity and frequency in activating these pathways may be developed, allowing for the refinement of ES protocols.

Where possible, researchers should be encouraged to develop any research protocols as RCTs, as control data gives an important and clear comparator for appropriate statistical analysis with matched participants.

The most important recommendation for future studies is to report negative and non-significant results. Unpublished negative results, or no data given for non-statistically significant results can be dangerous as it can bias meta-analyses to report on available data, which is more likely to report on positive results (71).

## Conclusion

This systematic review and meta-analysis found that TENS, TSCS, FES cycling and FES gait were effective at reducing spasticity in people with SCI, according to MAS scores, for both RCT and non-RCT studies, and R_2n_ values in non-RCT studies. There was little data available to assess how these results may correlate with neurophysiological measures; however, results from two studies showed some similar implications for spasticity from both MAS scores and H_max_/M_max_ ratios.

Reduction in spasticity following sub-MT TSCS and TENS indicate that activation of afferent fibres is at least required for ES to be an effective intervention for spasticity management in people with SCI. Pairing ES with functional movement may not be necessary, however results do not show a negative effect on spasticity in this case.

This review concludes that more evidence is required for specific stimulation parameters to be recommended (frequency, pulse width and stimulation dose) and for the effects of these parameters on varying levels and severities of SCI.

## Data Availability

The raw data supporting the conclusions of this article will be made available by the authors, without undue reservation.

## References

[1] World Health Organisation. Spinal Cord Injury (2013). Available at: https://www.who.int/news-room/fact-sheets/detail/spinal-cord-injury

[2] American Association of Neurological Surgeons. Spasticity (2019). Available at: https://www.aans.org/Patients/Neurosurgical-Conditions-and-Treatments/Spasticity (cited 2019 Jun 14).

[3] Holtz KA Lipson R Noonan VK Kwon BK Mills PB. Prevalence and effect of problematic spasticity after traumatic spinal cord injury. Arch Phys Med Rehabil. (2017) 98(6):1132–8. 10.1016/j.apmr.2016.09.124 27780743

[4] Sangari S Perez MA. Prevalence of spasticity in humans with spinal cord injury with different injury severity. J Neurophysiol. (2022) 128(3):470–9. 10.1152/jn.00126.2022 35507475 PMC9423778

[5] University College London Hospitals. Trust Nhs planning/Lit review notes/uclh spasticity management 2019. pd. F. Spasticity management (2018). Available at: https://www.uclh.nhs.uk/OurServices/ServiceA-Z/Neuro/SPAS/Pages/Home.aspx (cited 2019 Mar 25).

[6] Levy J Molteni F Cannaviello G Lansaman T Roche N Bensmail D. Does botulinum toxin treatment improve upper limb active function? Ann Phys Rehabil Med. (2018) 62(4):1–7. Available at: https://linkinghub.elsevier.com/retrieve/pii/S187706571831409X. 10.1016/j.rehab.2018.05.1320 29960017

[7] Deltombe T Lejeune T Gustin T. Botulinum toxin type a or selective neurotomy for treating focal spastic muscle overactivity? Ann Phys Rehabil Med. (2018) 62(4):220–24. 10.1016/j.rehab.2018.07.008 30107243

[8] Adams MM Hicks AL. Spasticity after spinal cord injury. Spinal Cord. (2005) 43(10):577–86. 10.1038/sj.sc.3101757 15838527

[9] National Institue for Health and Care Excellence. RT300 For spinal cord injury rehabilitation. (2019). Available at: https://www.nice.org.uk/advice/mib169/resources/rt300-for-spinal-cord-injury-rehabilitation-pdf-2285963585494981

[10] National Institute for Health and Care Excellence. Senza spinal cord stimulation system for delivering HF10 therapy to treat chronic neuropathic pain. (2019). Available at: https://www.nice.org.uk/guidance/mtg41

[11] National Institute for Health and Care Excellence. Spinal cord stimulation for chronic pain of neuropathic or ischaemic origin: Systematic review and economic evaluation. Health Technology Assessment (Rockv) (2009). Vol. 13. 10.3310/hta13170 19331797

[12] National Institute for Health and Care Excellence. Evoke spinal cord stimulator for managing chronic neuropathic or ischaemic pain. (2020). Available at: https://www.nice.org.uk/advice/mib238/resources/evoke-spinal-cord-stimulator-for-managing-chronic-neuropathic-or-ischaemic-pain-pdf-2285965579199173

[13] Faist M Mazevet D Dietz V Pierrot-deseilligny E. A quantitative assessment of presynaptic inhibition of ia afferents in spastics. Difference in hemiplegics and paraplegics. Brain. (1994) 117(6):1449–55. 10.1093/brain/117.6.1449 7820579

[14] Aymard C Katz R Lafitte C Lo E Pénicaud A Pradat-Diehl P Presynaptic inhibition and homosynaptic depression: a comparison between lower and upper limbs in Normal human subjects and patients with hemiplegia. Brain. (2000) 123(Pt 8):1688–702. 10.1093/brain/123.8.1688 10908198

[15] Angel RW Hofmann WW. The H reflex in Normal, spastic, and rigid subjects. Arch Neurol. (1963) 8(6):591–6. 10.1001/archneur.1963.00460060021002 14059331

[16] Cakar E Akyuz G Durmus O Bayman L Yagci I Karadag-Saygi E The relationships of motor-evoked potentials to hand dexterity, motor function, and spasticity in chronic stroke patients: a transcranial magnetic stimulation study. Acta Neurol Belg. (2016) 116(4):481–7. 10.1007/s13760-016-0633-2 27037821

[17] Sangari S Lundell H Kirshblum S Perez MA. Residual descending motor pathways influence spasticity after spinal cord injury. Ann Neurol. (2019) 86(305):ana.25505. 10.1002/ana.25505 PMC6786768 31102289

[18] Mills PB Dossa F. Transcutaneous electrical nerve stimulation for management of limb spasticity: a systematic review. Am J Phys Med Rehabil. (2016) 95(4):309–18. 10.1097/PHM.0000000000000437 26829077

[19] Luo S Xu H Zuo Y Liu X All AH. A review of functional electrical stimulation treatment in spinal cord injury. NeuroMolecular Med. (2020) 22(4):447–63 (0123456789). 10.1007/s12017-019-08589-9 31916220

[20] Bekhet AH Bochkezanian V Saab IM Gorgey AS. The effects of electrical stimulation parameters in managing spasticity after spinal cord injury: a systematic review. Am J Phys Med Rehabil. (2019) 98(6):484–99. 10.1097/PHM.0000000000001064 30300228

[21] Fernández-Tenorio E Serrano-Muñoz D Avendaño-Coy J Gómez-Soriano J. Transcutaneous electrical nerve stimulation for spasticity: a systematic review. Neurol. (2016) 34(7):451–60. 10.1016/j.nrleng.2018.08.001 27474366

[22] Mahmood A Veluswamy SK Hombali A Mullick A NM Solomon JM. Effect of transcutaneous electrical nerve stimulation on spasticity in adults with stroke: a systematic review and meta-analysis. Arch Phys Med Rehabil. (2019) 100(4):751–68. 10.1016/j.apmr.2018.10.016 30452892

[23] Nagel SJ Wilson S Johnson MD Machado A Frizon L Chardon MK Spinal cord stimulation for spasticity: historical approaches, current Status, and future directions. Neuromodulation Technol Neural Interface. (2017) 20(4):307–21. 10.1111/ner.12591 28370802

[24] Fang CY Lien ASY Tsai JL Yang HC Chan HL Chen RS The effect and dose-response of functional electrical stimulation cycling training on spasticity in individuals with spinal cord injury: a systematic review with meta-analysis. Front Physiol. (2021) 12(November):1–14. 10.3389/fphys.2021.756200 PMC8640241 34867459

[25] Higgins J Thomas J Chandler J Cumpston M Li T Page M Cochrane handbook for systematic reviews of interventions. Cochrane (2020). Available at: www.training.cochrane.org/handbook (cited 2020 Sep 22).

[26] Massey SJ. Neuromodulation in the treatment of upper limb spasticity. University College London (UCL) (2021).

[27] Physiotherapist Evidence Database. PEDro scale. (1999). Available at: https://pedro.org.au/english/resources/pedro-scale/ (cited 2020 Oct 1).

[28] Bogart S. SankeyMATIC. (2022). Available at: https://sankeymatic.com/build/ (cited 2022 Sep 7).

[29] Aydin G Tomruk S Keleş I Demir SÖO Orkun S Keles I Transcutaneous electrical nerve stimulation versus baclofen in spasticity: clinical and electrophysiologic comparison. Am J Phys Med Rehabil. (2005) 84(8):584–92. 10.1097/01.phm.0000171173.86312.69 16034227

[30] Bajd T Gregoric M Vodovnik L Benko H. Electrical stimulation in treating spasticity resulting from spinal cord injury. Arch Phys Med Rehabil. (1985) 66(8):515–7. Available at: https://www.scopus.com/inward/record.uri?eid=2-s2.0-0021797524&partnerID=40&md5=b4815173785bcc0fd04f9994ad151d67. 10.5555/uri:pii:000399938591247X 3875331

[31] Oo WM. Efficacy of addition of transcutaneous electrical nerve stimulation to standardized physical therapy in subacute spinal spasticity: a randomized controlled trial. Arch Phys Med Rehabil. (2014) 95(11):2013–20. 10.1016/j.apmr.2014.06.001 24953249

[32] Franek A Turczynski B Opara J. Treatment of spinal spasticity by electrical stimulation. J Biomed Eng. (1988) 10(3):266–70. 10.1016/0141-5425(88)90009-X 3260641

[33] Goulet C Arsenault AB Bourbonnais D Laramee M-TT Lepage Y. Effects of transcutaneous electrical nerve stimulation on H-reflex and spinal spasticity. Scand J Rehabil Med. (1996) 28(3):169–76. Available at: https://www.scopus.com/inward/record.uri?eid=2-s2.0-002976307 8885040

[34] Khanna S Kaur J. Comparison of agonist vs. Antagonist stimulation on triceps surae spasticity in spinal cord injury. Iran Rehabil J. (2017) 15(2):117–24. 10.18869/nrip.irj.15.2.117

[35] Perdan J Kamnik R Ceru B Bajd T Savrin R Jelenc J Comparison of four evaluation approaches in transcutaneous electrical nerve stimulation treatment in two incomplete tetraplegic subjects. Neuromodulation. (2010) 13(3):238–45. 10.1111/j.1525-1403.2009.00266.x 21992839

[36] Robinson CJ Kett NA Bolam JM. Spasticity in spinal cord injured patients: 1. Short-term effects of surface electrical stimulation. Arch Phys Med Rehabil. (1988) 69(8):598–604. Available at: https://www.scopus.com/inward/record.uri?eid=2-s2.0-0023719551&partnerID=40&md5=f045afa6e2e148a6a197bfc0ca2a235d 3261577

[37] Sivaramakrishnan A Solomon JM Manikandan N Solomon JM Manikandan N. Comparison of transcutaneous electrical nerve stimulation (TENS) and functional electrical stimulation (FES) for spasticity in spinal cord injury—a pilot randomized cross-over trial. *J Spinal Cord Med.* (2018) 41(4):397–406. 10.1080/10790268.2017.1390930 PMC6055976 29067867

[38] Van Der Salm A Veltink PH Hermens HJ Nene AV Ijzerman MJ. Effect of electrical stimulation of hamstrings and L3/4 dermatome on gait in spinal cord injury. Neuromodulation. (2006) 9(1):48–55. 10.1111/j.1525-1403.2006.00042.x 22151593

[39] Vodovnik L Bowman BR Hufford P. Effects of electrical stimulation on spinal spasticity. Scand J Rehabil Med. (1984) 16(1):29–34. Available at: https://www.cochranelibrary.com/central/doi/10.1002/central/CN-00652157/full 6608787

[40] Estes SP Iddings JA Field-Fote EC. Priming neural circuits to modulate spinal reflex excitability. Front Neurol. (2017) 8(FEB):1–10. Available at: file:///C:/Users/DELL/AppData/Local/Mendeley Ltd./Mendeley Desktop/Downloaded/Estes, Iddings, Field-Fote - 2017 - Priming neural circuits to modulate spinal reflex excitability.pdf. 10.3389/fneur.2017.00017 28217104 PMC5289977

[41] Estes S Zarkou A Hope JM Suri C Field-Fote EC. Combined transcutaneous spinal stimulation and locomotor training to improve walking function and reduce spasticity in subacute spinal cord injury: a randomized study of clinical feasibility and efficacy. J Clin Med. (2021) 10(6):1–17. 10.3390/jcm10061167 PMC7999894 33799508

[42] Hofstoetter MW Tansey KE Mayr W Kern H Minassian K. Modification of spasticity by transcutaneous spinal cord stimulation in individuals with incomplete spinal cord injury. J Spinal Cord Med. (2014) 37(2):202–11. 10.1179/2045772313Y.0000000149 24090290 PMC4066429

[43] Hofstoetter US Freundl B Danner SM Krenn MJ Mayr W Binder H Transcutaneous spinal cord stimulation induces temporary attenuation of spasticity in individuals with spinal cord injury. J Neurotrauma. (2020) 37(3):481–93. 10.1089/neu.2019.6588 31333064

[44] Vargas Luna JL Guðfinnsdóttir HK Magnúsdóttir G Guðmundsdóttir V Krenn M Mayr W Effects of sustained electrical stimulation on spasticity assessed by the pendulum test. Curr Dir Biomed Eng. (2016) 2(1):405–7. 10.1515/cdbme-2016-0090

[45] Sandler EB Condon K Field-Fote EC. Efficacy of transcutaneous spinal stimulation versus whole body vibration for spasticity reduction in persons with spinal cord injury. J Clin Med. (2021) 10(15):1–11. 10.3390/jcm10153267 PMC8348743 34362051

[46] Skold C Lonn L Harms-ringdahl K Hultling C Levi R Nash M Effects of functional electrical stimulation training for six months on body composition and spasticity in motor complete tetraplegic spinal cord-injured individuals. J Rehabil Med. (2002) 34(1):25–32. 10.1080/165019702317242677 11900259

[47] Krause P Szecsi J Straube A Munich AS Straube A Munich AS Changes in spastic muscle tone increase in patients with spinal cord injury using functional electrical stimulation and passive leg movements. Clin Rehabil. (2008) 22(7):627–34. 10.1177/0269215507084648 18586814

[48] Mazzoleni S Stampacchia G Gerini A Tombini T Carrozza MC. FES-cycling training in spinal cord injured patients. In: *2013 35th Annual International Conference of the IEEE Engineering in Medicine and Biology Society* (*EMBC*). Osaka, Japan: IEEE; (2013):539–41. Available at: file:///C:/Users/DELL/Downloads/06610755.pdf. 10.1109/EMBC.2013.6610755 24110942

[49] Ralston KE Harvey LA Batty J Lee BB Ben M Cusmiani R Functional electrical stimulation cycling has no clear effect on urine output, lower limb swelling, and spasticity in people with spinal cord injury: a randomised cross-over trial. J Physiother. (2013) 59(4):237–43. 10.1016/S1836-9553(13)70200-5 24287217

[50] Kuhn D Leichtfried V Schobersberger W. Four weeks of functional electrical stimulated cycling after spinal cord injury : a clinical cohort study. Int J Rehabil Res. (2004) 37(3):243–50. 10.1097/MRR.0000000000000062. Available at: file:///C:/Users/DELL/Downloads/Four_weeks_of_functional_electrical_stimulated.8.pdf. 24802976

[51] Mazzoleni S Battini E Rustici A Stampacchia G. An integrated gait rehabilitation training based on functional electrical stimulation cycling and overground robotic exoskeleton in complete spinal cord injury patients : preliminary results. Int Conf Rehabil Robot. (2017) 2017:0–4. Available at: file:///C:/Users/DELL/Downloads/08009261.pdf. 10.1109/icorr.2017.8009261 28813833

[52] Gant KL Nagle KG Cowan RE Field-Fote EC Nash MS Kressler J Body system effects of a multi-modal training program targeting chronic, motor complete thoracic spinal cord injury. J Neurotrauma. (2018) 35(3):411–23. 10.1089/neu.2017.5105 28795657 PMC6909697

[53] Duffell LD Paddison S Alahmary AF Donaldson N Burridge J. The effects of FES cycling combined with virtual reality racing biofeedback on voluntary function after incomplete SCI: a pilot study. J Neuroeng Rehabil. (2019) 16(1):1–15. Available at: internal-pdf://0002482911/Duffell-2019-The effects of FES cycling combin.pdf. 10.1186/s12984-019-0619-4 31771600 PMC6880599

[54] Granat MH Ferguson ACB Andrews BJ Delargy M. The role of functional electrical stimulation in the rehabilitation of patients with incomplete spinal cord injury—observed benefits during gait studies. (1993) 31:207–15. 10.1038/sc.1993.39 8493035

[55] Kapadia N Masani K Craven BC Giangregorio LM Hitzig SL Richards K A randomized trial of functional electrical stimulation for walking in incomplete spinal cord injury: effects on walking competency. J Spinal Cord Med. (2014) 37(5):511–24. 10.1179/2045772314Y.0000000263 25229735 PMC4166186

[56] Murray SA Farris RJ Golfarb M Hartigan C Truex D Kandilakis C FES coupled with A powered exoskeleton for cooperative muscle contribution in persons with paraplegia. Conf Proc IEEE Eng Med Biol Soc. (2018) 2018:2788–92. Available at: file:///C:/Users/DELL/Downloads/08512810.pdf. 10.1109/embc.2018.8512810 30440980

[57] Meyer C Hofstoetter US Hubli M Hassani RH Rinaldo C Curt A Pendulum testing of spasticity. J Biomed Eng. (1984) 6(1):9–16. 10.1016/0141-5425(84)90003-7 6694374

[58] Meyer C Hofstoetter US Hubli M Hassani RH Rinaldo C Curt A Immediate effects of transcutaneous spinal cord stimulation on motor function in chronic, sensorimotor incomplete spinal cord injury. J Clin Med. (2020) 9(11):1–18. 10.3390/jcm9113541 PMC7694146 33147884

[59] Vodovnik L Stefanovska A Bajd T. Effects of stimulation parameters on modification of spinal spasticity. Med Biol Eng Comput. (1987) 25(4):439–42. 10.1007/BF02443365 3502481

[60] Van Der SA Veltink PH Ijzerman MJ Van Der Salm A Veltink PH Ijzerman MJ Comparison of electric stimulation methods for reduction of triceps surae spasticity in spinal cord injury. Arch Phys Med Rehabil. (2006) 87(2):222–8. 10.1016/j.apmr.2005.09.024 16442976

[61] Thompson AK Lapallo B Duffield M Abel BM Pomerantz F. Repetitive common peroneal nerve stimulation increases ankle dorsiflexor motor evoked potentials in incomplete spinal cord lesions. Exp Brain Res. (2011) 210(1):143–52. 10.1007/s00221-011-2607-1 21360230

[62] Robinson CJ Kett NA Bolam JM. Spasticity in spinal cord injured patients: 2. Initial measures and long-term effects of surface electrical stimulation. Arch Phys Med Rehabil. (1988) 69(10):862–8. Available at: https://www.scopus.com/inward/record.uri?eid=2-s2.0-0023798502&partnerID=40&md5=6924f17aaeadb8420a91f5b4cedc4a8b 3263102

[63] Pierrot-Deseilligny E Burke D. The pathophysiology of spasticity and parkinsonian rigidity. In: The circuitry of the human spinal cord. (2009). p. 556–600. Available at: https://www.scopus.com/inward/record.uri?eid=2-s2.0-84949321571&doi=10.1038%2Fsc.2015.19&partnerID=40&md5=c1e2e0f831215246809c49d753 36cf33174

[64] Yasąr E Ylmaz B Göktepe S Kesikburun S Yaşar E Yılmaz B The effect of functional electrical stimulation cycling on late functional improvement in patients with chronic incomplete spinal cord injury. Spinal Cord. (2015) 53(12):866–9. 10.1038/sc.2015.19 25687513

[65] Pierrot-Deseilligy E Burke D. The circuitry of the human spinal cord. 1st ed. Cambridge University Press (2012).

[66] Mima T Oga T Rothwell J Satow T Yamamoto JI Toma K Short-term high-frequency transcutaneous electrical nerve stimulation decreases human motor cortex excitability. Neurosci Lett. (2004) 355(1–2):85–8. 10.1016/j.neulet.2003.10.045 14729241

[67] Knikou M. The H-reflex as a probe: pathways and pitfalls. J Neurosci Methods. (2008) 171(1):1–12. 10.1016/j.jneumeth.2008.02.012 18394711

[68] Krause P Szecsi J Straube A. FES Cycling reduces spastic muscle tone in a patient with multiple sclerosis. NeuroRehabilitation. (2007) 22(4):335–7. 10.3233/NRE-2007-22412 17971625

[69] Dietz V. Spinal cord pattern generators for locomotion. Clin Neurophysiol. (2003) 114(8):1379–89. 10.1016/S1388-2457(03)00120-2 12888019

[70] Perez MA Field-Fote EC Floeter MK. Patterned sensory stimulation induces plasticity in reciprocal ia inhibition in humans. J Neurosci. (2003) 23(6):2014–8. 10.1523/JNEUROSCI.23-06-02014.2003 12657659 PMC6742007

[71] Mlinaric A Horvat M Smolcic V. Dealing with the positive publication bias: why you should really publish your negative results. Biochem Med. (2017) 27(3):1–6. 10.11613/BM.2017.030201 PMC5696751 29180912

