# Supplementary material for: Neurophysiological and clinical outcome measures of the impact of electrical stimulation on spasticity in spinal cord injury: Systematic review and meta-analysis
Source: Front Rehabil Sci. 2022 Dec 16;3:1058663. doi: 10.3389/fresc.2022.1058663 (PMC9801305; doi:10.3389/fresc.2022.1058663)
Supplement: Supplementary file 1 [file Table1.docx]

Supplementary Material

**
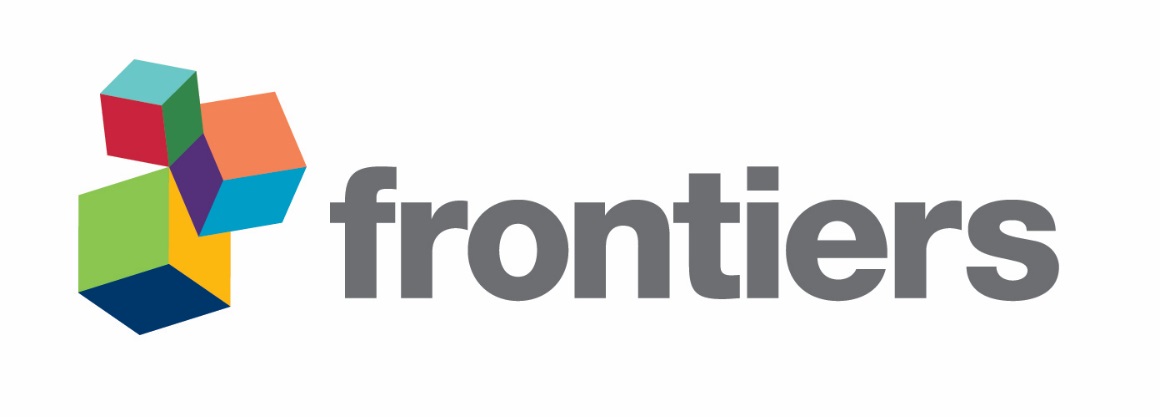
**

.

Supplementary Table 1: Methodological quality assessment of randomised trials included in the systematic review according to the PEDro scale. Total scores are out of a possible 11 points

|  | Aydin 2005 | Estes 2017 | Estes 2021 | | Franek 1988 | | Kapadia 2014 | | Khanna 2017 | Krause 2008 | Oo 2014 | Ralston 2013 | Sivara-makris-hnan 2018 | Van der Salm 2006 | | Vodovnik 1987 | |
| --- | --- | --- | --- | --- | --- | --- | --- | --- | --- | --- | --- | --- | --- | --- | --- | --- | --- |
| Eligibility criteria specified |  | ✓ | | ✓ | |  | | ✓ | ✓ | ✓ | ✓ | ✓ | ✓ | | ✓ | |  |
| Random allocation | ✓ | ✓ | | ✓ | |  | | ✓ | ✓ |  | ✓ | ✓ | ✓ | |  | |  |
| Concealed allocation |  |  | |  | |  | | ✓ |  |  | ✓ | ✓ | ✓ | |  | |  |
| Groups similar at baseline (in terms of important prognostic indicators) | ✓ | ✓ | |  | | ✓ | | ✓ | ✓ | ✓ | ✓ | ✓ | ✓ | | ✓ | | ✓ |
| All subjects were blinded |  |  | |  | |  | |  |  |  |  |  | ✓ | |  | |  |
| Therapists who administered intervention blinded |  |  | |  | |  | |  |  |  |  |  |  | |  | |  |
| All assessors blinded |  |  | |  | |  | | ✓ |  |  | ✓ | ✓ | ✓ | |  | |  |
| ≥1 outcome measure obtained from >85 % of initially allocated participants | ✓ |  | | ✓ | |  | |  | ✓ | ✓ | ✓ | ✓ | ✓ | | ✓ | | ✓ |
| Intervention was given as allocated |  |  | | ✓ | | ✓ | |  | ✓ | ✓ | ✓ | ✓ | ✓ | | ✓ | | ✓ |
| Between-group statistics performed | ✓ | ✓ | | ✓ | |  | | ✓ | ✓ | ✓ | ✓ | ✓ | ✓ | | ✓ | |  |
| Point measures and variability assessed | ✓ | ✓ | | ✓ | |  | | ✓ | ✓ | ✓ |  | ✓ |  | | ✓ | | ✓ |
| Total score | **5** | **5** | | **6** | | **2** | | **7** | **7** | **6** | **8** | **9** | **9** | | **6** | | **4** |

Supplementary Table 2: Main characteristics of included studies. Abbreviations: AS = Ashworth scale, CSS = composite spasticity score, DF = dorsiflexors, DTR = deep tendon reflex, EMG = electromyography, FDS = functional disability score, FES = functional electrical stimulation, FIM = functional independence measure, GL = gluteals, HA, = hamstrings, HTI = highest tolerated intensity, MAS = modified Ashworth scale, MT = motor threshold, NR = not reported, PF = plantar-flexors, PT = pendulum test, QU = quadriceps, SCATS = spinal cord assessment tool for spastic reflexes, SFS = spasm frequency scale, ST = sensory threshold, TA = tibialis anterior, TS = triceps surae, TUGT = timed up-and-go test, VAS = visual analogue scale, 6MWT = 6-minute walking test. ✓ denotes an improvement in the outcome measure, ꓳ denotes no change and ✘ denotes a worsening, * denotes a statistically significant result, unless stated otherwise.

| Study | Study design | N = | AIS | Time since injury | Form of ES | Stimulat-ion location | Freq. (Hz) | Pulse width (ms) | Intensity | Duration | Result |
| --- | --- | --- | --- | --- | --- | --- | --- | --- | --- | --- | --- |
| Aydin (2005) | Pre-post | 10 SCI, 20 healthy | A |  | TENS | Bilateral tibial nerves | 100 | 100 | 50 mA | 15 mins/dy for 15 dy | **✓*** AS, SFS*,* DTR, FDS, FIM Most improvement seen following 15th session of TENS  **✓*** Hmax |
| Bajd (1985) | Pre-post | 6 |  |  | TENS | Above and below the knee | 100 | 0.3 | ≤ 50 mA, < MT | 20 mins | **✓*** PT |
| Duffell (2019) | Pre-post | 11 | C-D | 2 mnth-49 yr | FES cycling | QUs, HA, GL | 30 | 0.2 | Gradual-ly increased from minim-um to HTI | 20-45 minutes, 3x/ wk over 4 wk | **✓** MAS  **✓*** Voluntary power output, motor scores |
| Estes (2017) | Random-ised crossov-er | 10 | B-D | >2 yr | TSCS | T11/T12 | 50 | NR | Produci-ng paraesth-esia/HTI | 30 mins | **✓*** PT for 45 minutes following TSCS |
| Estes (2021) | RCT | 16 |  | 2-6 mnth | TSCS | T11/T12 | 50 | NR | Produci-ng paraesth-esia/HTI | 30 mins, 3x/ wk for 2 wks | **✓** improvements in PT for both groups  **✓*** improvements in walking outcomes in TSCS group |
| Franek (1988) | RCT | 44 ES, 35 control | A-D | 6-96 mnth | TENS | Hip abductors, anterior thigh, GL | 5 to 7 | NR | 10-15 V | 18x for 2.5 min/dy for 6 dy, 2x/dy for 6 dy | **✓** PT, own spasticity scale Reduction in PT more marked in TENS group than in control group |
| Gant (2018) | Pre-post | 8 | A/B | ≥1 yr | FES cycling |  | 35 | 0.35 | 100-140 mA | 12 wk | ✘ MAS increased in most participants, or was unchanged  **ꓳ** PT and H/M ratio varied between participants |
| Goulet (1996) | Pre-post | 14 | A-D | 2-194 mnth | TENS | TS | 99 | 0.25 | 15 mA (2x ST in healthy particip-ants) | 30 mins | **✓*** MAS, Achilles DTR, (clonus - non-significant)  **ꓳ** H-reflex |
| Granat (1993) | Pre-post | 6 | C/D | ≥ 2 yr | FES gait | QU, hip abductors, HA, erector spinae | 25 | 0.3 | NR | 30 mins/dy, <5 dy/wk for 6 mnth | **✓*** PT  **ꓳ** AS varied between participants |
| Hofstoett-er (2020) | Pre-post | 12 | A, C, D | ≥ 1 yr | TSCS | T11/T12 | 50 | 1 | 16-100 mA, Sub-PRR threshold | 30 mins. 6-week protocol in 1 participa-nt. 30mins/ dy, 4 dys/wk | **✓*** MAS, PT immediately after and 2 hours after TSCS  **✓*** Clonus, cutaneous-input evoked spasms, passive joint movement immediately after and 2 hours after TSCS  **ꓳ** 10 m walk test |
| Hofstoett-er (2014) | Pre-post | 3 | D | 9-12 yr | TSCS | T11/T12 | 50 | 1 | Produci-ng paraesth-esias, < MT | 30 mins | **✓** PT*,* stretch reflex |
| Kapadia (2014) | Random-ised control trial | 16 | C-D | ≥ 1.5 yr | FES gait | QU, HA, DF, PF | 40 | 0-0.3 | 8-125 mA, > MT | 45 mins per session, 3 dy/wk, 16 wk | **ꓳ** MAS, PT, SCIM over time, or between intervention groups |
| Khanna (2017) | Crossov-er trial | 30 | A-D | >6 mnth | TENS | TS, TA | 30 | 0.3 | At MT, or just below is spasms occurred | 20 mins, 5x/wk for 2 wks | **✓*** MAS, DTR  No difference between stimulation groups  **ꓳ** Clonus |
| Krause (2008) | Crossov-er trial with passive cycling | 5 | A | 3-9 yr | FES cycling | QU, HA, glutes | 20 | 0.5 | 0-99 mA | 60-100 mins | **✓*** MAS, PT MAS reduced after both FES and passive cycling sessions |
| Kuhn (2014) | Pre-post | 30 | A-D | < 4 wk-122 mnth | FES cycling | QU, HA, GL | 30 | 0.25 | 10-130 mA | 20 mins 2 dy/wk for 4 wks | **✓*** MAS, muscle circumference (non-significant for AIS A & B) |
| Mazzoleni (2013) | Pre-post | 20 | A-C |  | FES cycling | QU, femoral biceps | NR | 0.05-0.5 | Up to 140 mA | 20 sessions, 3/wk | **ꓳ** MAS, PSFS, SCIM |
| Mazzoleni (2017) | Pre-post | 7 | A |  | FES cycling | QUs, femoral biceps, GL | 50 | 0.5 | Quads: 35-75mA, femoral biceps: 25-50mA | 20 sessions, 3/wk | **✓*** MAS, 6MWT, TUGT, standing time, number of steps (PSFS - non-significant) |
| Murray (2018) | Pre-post | 3 | A and C |  | FES gait | QU, GL, HA, TA, trunk | 40 | 0.2-0.35 | 22-70 mA | 15-25 mins | **✓** MAS |
| Oo (2014) | RCT | 8 | A-D | ~2-4 mnth | TENS | Common peroneal nerve | 100 | 0.1-0.3 | 15 mA (2x ST in healthy participants) | 60 mins | **✓*** CSS and clonus score in the experimental group compared to the control |
| Perdan (2010) | Case studies | 2 | C, D | 3 and 4 mnth | TENS | Whole hand | 50 | 0.2 | ST | 20 mins, 5 dy/wk for 4 wk | **✓** Strength, motor control, hand function  **ꓳ** MAS |
| Ralston (2013) | Random-ised crossov-er | 14 | A-C | 64-135 dy | FES cycling | QU, HA, GL | 33 | NR | ≤ 140 mA | 30-45 mins, 4x/wk for 2 wk | **ꓳ** AS, no clear difference between FES cycling and standard rehabilitation |
| Robinson (1988) | Pre-post | 31 (8 for 8 wks) | A-D | 15 < 1 yr, 16 > 1 yr | TENS | QU | 20 | 0.4 | 120-160 mA | 20 mins, 2x/dy 6 dy/wk for 4-8 wks | **ꓳ**/✘ PT in most participants, which decreased by week 8 |
| Sandler (2021) | Random-ised crossov-er | 32 | C, D | > 6 mnth | TSCS | T11-T12 | 50 | 0.4 | Produci-ng paraesth-esias, < MT | 15 mins | **ꓳ** PT unchanged from baseline overall  **✓*** PT in those with severe spasticity |
| Sivaram-krishnan (2018) | Double blind random-ised crossov-er | 10 | A-E | 1-26 mnth | TENS and FES | TENS: QUs, adductors, PF. FES: QU, adductors, PF | TENS: 100 Hz, FES: 35 Hz | 0.3 | TENS: ≤ 20 mA, < MT  FES: 3x MT | 30 mins | **✓*** MAS, SCATS for up to 4 hours  No difference found between TENS and FES interventions |
| Skold (2002) | Pre-post | 15 |  | > 1 yr | FES cycling | Quads, hams, glutes | 60 | 0.35 | ≤ 130 mA, > MT | NR | **ꓳ** MAS, VAS  **ꓳ** EMG activity |
| Van der Salm (2006) | Placebo-controll-ed crossov-er | 10 | A and C | 28-275 mnth | TENS | TS, TA, or S1 dermatome (lateral side of the foot) | 30 | TS, TA stim: 0.03 ms, S1: 0.01 ms | TS and TA stim: 300 % MT, S1 stim: 80 % MT | 45 mins | **✓*** MAS Statistically significant differences found between TENS and placebo group  **ꓳ** H/M ratio |
| Vargas Luna (2016) | Pre-post | 4 | B-D | > 1 yr | TSCS | T11-T12 | 50 | 1 | 90 % of min. MT in all muscle groups | 30 mins | **✓** PT in 2 out of 3 the participants who presented with spasticity |
| Vodovnik (1987) | Compari-son crossov-er | 7 | A | 4-60 mnth | TENS | QU | 100, 100, 100, 1000, 1000, 10, 10, 10 | 1, 0.1, 0.01, 0.1, 0.01, 1, 0.1, 0.01 | ≤ 30 mA, > MT | 4s on 4s off for 20 mins | **✓** PT for all but 1 participant. 100 Hz was most effective at improving PT at various pulse widths  **✓** EMG activity |
| Yasar (2015) | Pre-post | 10 | C-D | > 2 yr | FES cycling | QU, HA, GL | 20 | 0.25 | 10-140 mA, > MT | 1 hour, 3x/week for 16 weeks | **✓*** MAS, FIM compared to baseline after 3-month intervention and at 3-month follow-up |
